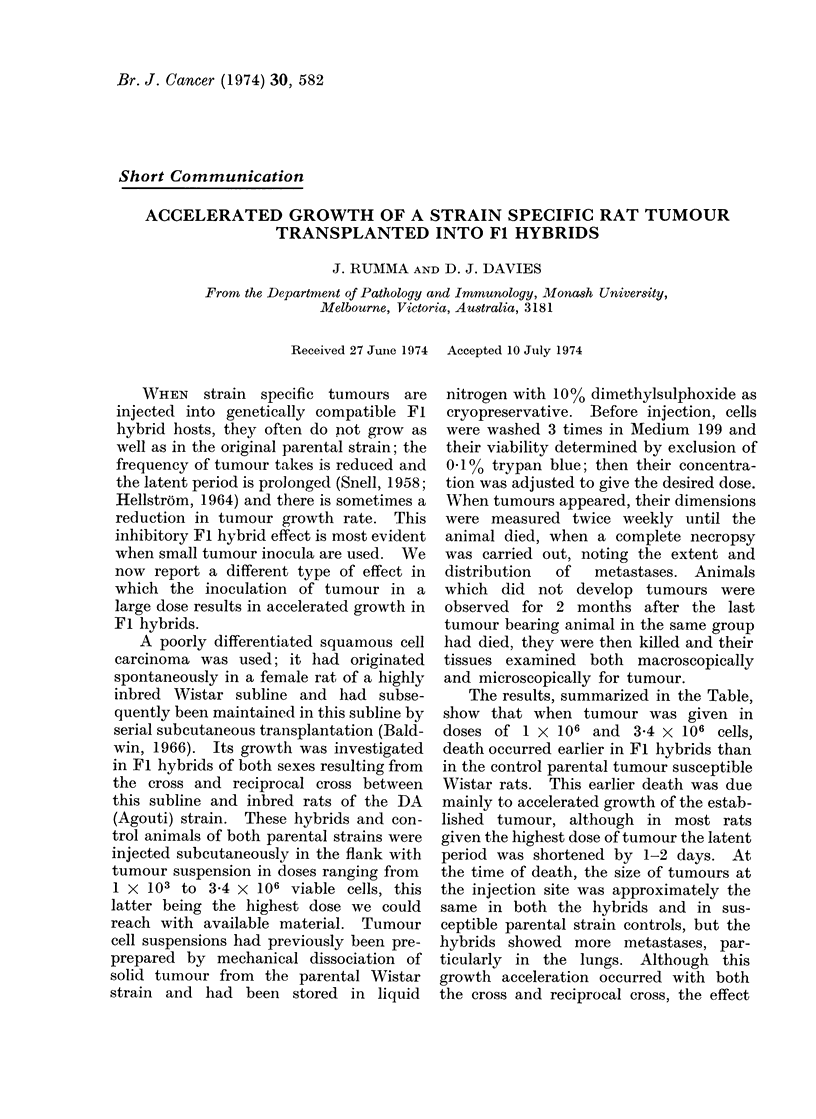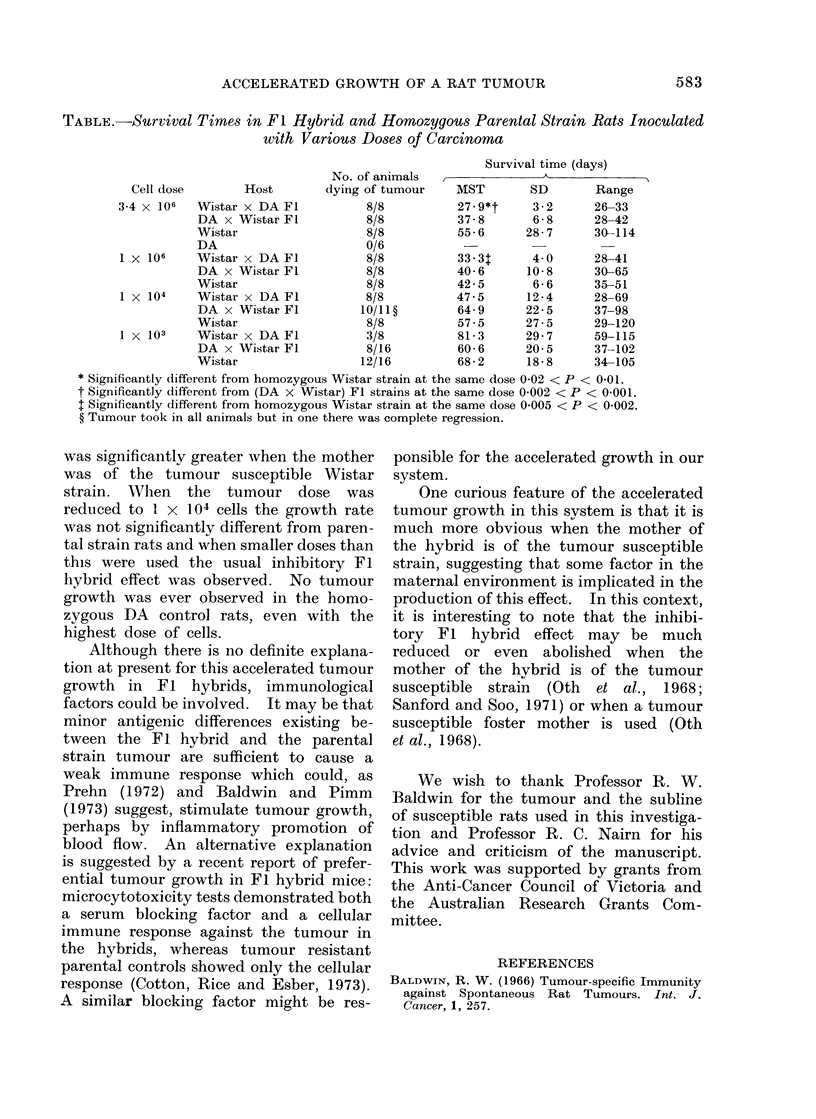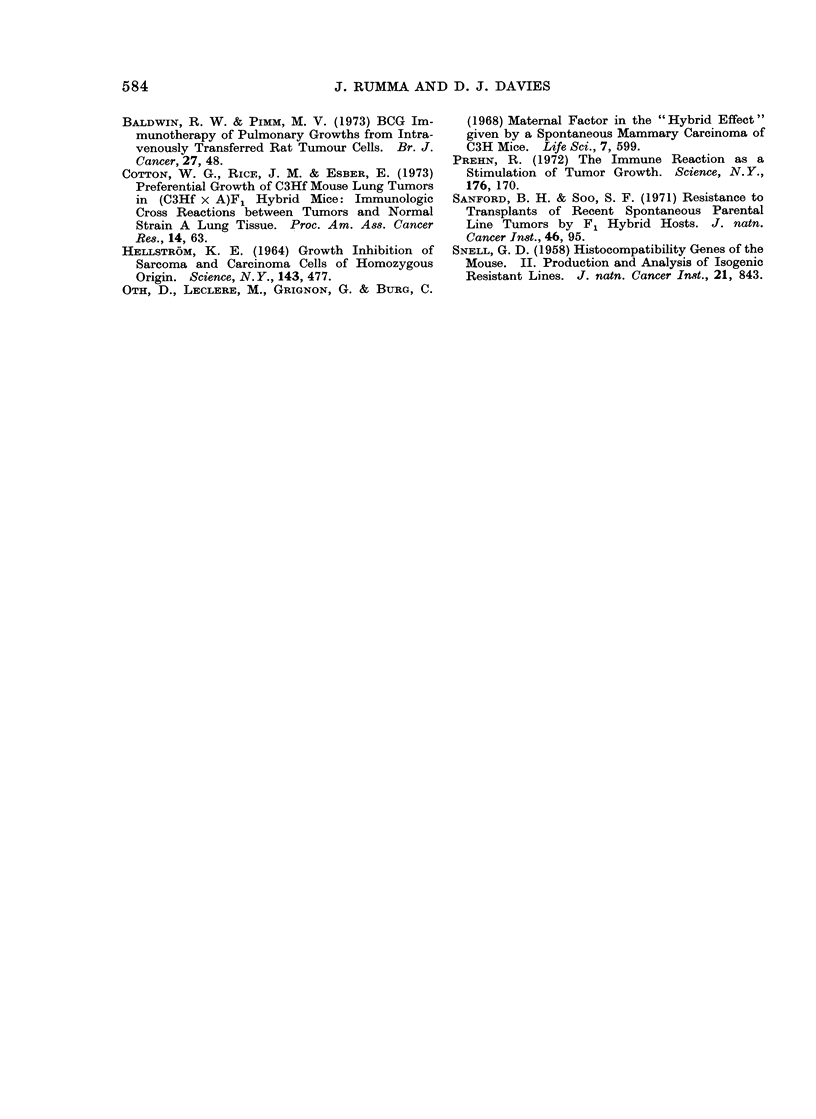# Accelerated growth of a strain specific rat tumour transplanted into F1 hybrids.

**DOI:** 10.1038/bjc.1974.239

**Published:** 1974-12

**Authors:** J. Rumma, D. J. Davies


Br. J. Cancer (1974) 30, 582

***Short Communication***

### ACCELERATED GROWTH OF A STRAIN SPECIFIC RAT TUMOUR TRANSPLANTED INTO F1 HYBRIDS

J. RUMMA AND D. J. DAVIES

*From the Department of Pathology and Immunology, Monash University, Melbourne, Victoria, Australia, 3181*

Received 27 June 1974 Accepted 10 July 1974

WHEN strain specific tumours are injected into genetically compatible F1 hybrid hosts, they often do not grow as well as in the original parental strain; the frequency of tumour takes is reduced and the latent period is prolonged (Snell, 1958; Hellström, 1964) and there is sometimes a reduction in tumour growth rate. This inhibitory F1 hybrid effect is most evident when small tumour inocula are used. We now report a different type of effect in which the inoculation of tumour in a large dose results in accelerated growth in F1 hybrids.

A poorly differentiated squamous cell carcinoma was used; it had originated spontaneously in a female rat of a highly inbred Wistar subline and had subsequently been maintained in this subline by serial subcutaneous transplantation (Baldwin, 1966). Its growth was investigated in F1 hybrids of both sexes resulting from the cross and reciprocal cross between this subline and inbred rats of the DA (Agouti) strain. These hybrids and control animals of both parental strains were injected subcutaneously in the flank with tumour suspension in doses ranging from $1 \times 10^3$ to $3{\cdot}4 \times 10^6$ viable cells, this latter being the highest dose we could reach with available material. Tumour cell suspensions had previously been pre-prepared by mechanical dissociation of solid tumour from the parental Wistar strain and had been stored in liquid nitrogen with 10% dimethylsulphoxide as cryopreservative. Before injection, cells were washed 3 times in Medium 199 and their viability determined by exclusion of 0·1% trypan blue; then their concentration was adjusted to give the desired dose. When tumours appeared, their dimensions were measured twice weekly until the animal died, when a complete necropsy was carried out, noting the extent and distribution of metastases. Animals which did not develop tumours were observed for 2 months after the last tumour bearing animal in the same group had died, they were then killed and their tissues examined both macroscopically and microscopically for tumour.

The results, summarized in the Table, show that when tumour was given in doses of $1 \times 10^6$ and $3{\cdot}4 \times 10^6$ cells, death occurred earlier in F1 hybrids than in the control parental tumour susceptible Wistar rats. This earlier death was due mainly to accelerated growth of the established tumour, although in most rats given the highest dose of tumour the latent period was shortened by 1–2 days. At the time of death, the size of tumours at the injection site was approximately the same in both the hybrids and in susceptible parental strain controls, but the hybrids showed more metastases, particularly in the lungs. Although this growth acceleration occurred with both the cross and reciprocal cross, the effect

TABLE.—*Survival Times in F1 Hybrid and Homozygous Parental Strain Rats Inoculated with Various Doses of Carcinoma*

| Cell dose | Host | No. of animals dying of tumour | Survival time (days) MST | SD | Range |
|---|---|---|---|---|---|
| $3\cdot4 \times 10^6$ | Wistar × DA F1 | 8/8 | 27·9*† | 3·2 | 26–33 |
| | DA × Wistar F1 | 8/8 | 37·8 | 6·8 | 28–42 |
| | Wistar | 8/8 | 55·6 | 28·7 | 30–114 |
| | DA | 0/6 | — | — | — |
| $1 \times 10^6$ | Wistar × DA F1 | 8/8 | 33·3‡ | 4·0 | 28–41 |
| | DA × Wistar F1 | 8/8 | 40·6 | 10·8 | 30–65 |
| | Wistar | 8/8 | 42·5 | 6·6 | 35–51 |
| $1 \times 10^4$ | Wistar × DA F1 | 8/8 | 47·5 | 12·4 | 28–69 |
| | DA × Wistar F1 | 10/11§ | 64·9 | 22·5 | 37–98 |
| | Wistar | 8/8 | 57·5 | 27·5 | 29–120 |
| $1 \times 10^3$ | Wistar × DA F1 | 3/8 | 81·3 | 29·7 | 59–115 |
| | DA × Wistar F1 | 8/16 | 60·6 | 20·5 | 37–102 |
| | Wistar | 12/16 | 68·2 | 18·8 | 34–105 |

* Significantly different from homozygous Wistar strain at the same dose $0{\cdot}02 < P < 0{\cdot}01$.
† Significantly different from (DA × Wistar) F1 strains at the same dose $0{\cdot}002 < P < 0{\cdot}001$.
‡ Significantly different from homozygous Wistar strain at the same dose $0{\cdot}005 < P < 0{\cdot}002$.
§ Tumour took in all animals but in one there was complete regression.

was significantly greater when the mother was of the tumour susceptible Wistar strain. When the tumour dose was reduced to $1 \times 10^4$ cells the growth rate was not significantly different from parental strain rats and when smaller doses than this were used the usual inhibitory F1 hybrid effect was observed. No tumour growth was ever observed in the homozygous DA control rats, even with the highest dose of cells.

Although there is no definite explanation at present for this accelerated tumour growth in F1 hybrids, immunological factors could be involved. It may be that minor antigenic differences existing between the F1 hybrid and the parental strain tumour are sufficient to cause a weak immune response which could, as Prehn (1972) and Baldwin and Pimm (1973) suggest, stimulate tumour growth, perhaps by inflammatory promotion of blood flow. An alternative explanation is suggested by a recent report of preferential tumour growth in F1 hybrid mice: microcytotoxicity tests demonstrated both a serum blocking factor and a cellular immune response against the tumour in the hybrids, whereas tumour resistant parental controls showed only the cellular response (Cotton, Rice and Esber, 1973). A similar blocking factor might be responsible for the accelerated growth in our system.

One curious feature of the accelerated tumour growth in this system is that it is much more obvious when the mother of the hybrid is of the tumour susceptible strain, suggesting that some factor in the maternal environment is implicated in the production of this effect. In this context, it is interesting to note that the inhibitory F1 hybrid effect may be much reduced or even abolished when the mother of the hybrid is of the tumour susceptible strain (Oth *et al.*, 1968; Sanford and Soo, 1971) or when a tumour susceptible foster mother is used (Oth *et al.*, 1968).

We wish to thank Professor R. W. Baldwin for the tumour and the subline of susceptible rats used in this investigation and Professor R. C. Nairn for his advice and criticism of the manuscript. This work was supported by grants from the Anti-Cancer Council of Victoria and the Australian Research Grants Committee.

### REFERENCES

BALDWIN, R. W. (1966) Tumour-specific Immunity against Spontaneous Rat Tumours. *Int. J. Cancer*, **1**, 257.

BALDWIN, R. W. & PIMM, M. V. (1973) BCG Immunotherapy of Pulmonary Growths from Intravenously Transferred Rat Tumour Cells. *Br. J. Cancer*, **27**, 48.

COTTON, W. G., RICE, J. M. & ESBER, E. (1973) Preferential Growth of C3Hf Mouse Lung Tumors in (C3Hf × A)$F_1$ Hybrid Mice: Immunologic Cross Reactions between Tumors and Normal Strain A Lung Tissue. *Proc. Am. Ass. Cancer Res.*, **14**, 63.

HELLSTRÖM, K. E. (1964) Growth Inhibition of Sarcoma and Carcinoma Cells of Homozygous Origin. *Science, N.Y.*, **143**, 477.

OTH, D., LECLERE, M., GRIGNON, G. & BURG, C. (1968) Maternal Factor in the "Hybrid Effect" given by a Spontaneous Mammary Carcinoma of C3H Mice. *Life Sci.*, **7**, 599.

PREHN, R. (1972) The Immune Reaction as a Stimulation of Tumor Growth. *Science, N.Y.*, **176**, 170.

SANFORD, B. H. & SOO, S. F. (1971) Resistance to Transplants of Recent Spontaneous Parental Line Tumors by $F_1$ Hybrid Hosts. *J. natn. Cancer Inst.*, **46**, 95.

SNELL, G. D. (1958) Histocompatibility Genes of the Mouse. II. Production and Analysis of Isogenic Resistant Lines. *J. natn. Cancer Inst.*, **21**, 843.